# Disruption of Active Trans-Sialidase Genes Impairs Egress from Mammalian Host Cells and Generates Highly Attenuated Trypanosoma cruzi Parasites

**DOI:** 10.1128/mbio.03478-21

**Published:** 2022-01-25

**Authors:** Gabriela de A. Burle-Caldas, Nailma S. A. dos Santos, Júlia T. de Castro, Fernanda L. B. Mugge, Viviane Grazielle-Silva, Antônio Edson R. Oliveira, Milton C. A. Pereira, João Luís Reis-Cunha, Anderson Coqueiro dos Santos, Dawidson Assis Gomes, Daniella C. Bartholomeu, Nilmar S. Moretti, Sergio Schenkman, Ricardo T. Gazzinelli, Santuza M. R. Teixeira

**Affiliations:** a Departamento de Bioquímica e Imunologia, Universidade Federal de Minas Gerais, Belo Horizonte, Brazil; b Centro de Tecnologia em Vacinas, Universidade Federal de Minas Gerais, Belo Horizonte, Brazil; c Centro de Pesquisa Rene Rachou, Fundação Osvaldo Cruz, Belo Horizonte, Brazil; d Departamento de Parasitologia, Universidade Federal de Minas Gerais, Belo Horizonte, Brazil; e Departamento de Microbiologia, Imunologia e Parasitologia, Universidade Federal de São Paulo, Sao Paulo, Brazil; Harvard T. H. Chan School of Public Health

**Keywords:** *Trypanosoma cruzi*, trans-sialidase, virulence, vaccine, CRISPR-Cas9

## Abstract

Trans-sialidases (TS) are unusual enzymes present on the surface of Trypanosoma cruzi, the causative agent of Chagas disease. Encoded by the largest gene family in the T. cruzi genome, only few members of the TS family have catalytic activity. Active trans-sialidases (aTS) are responsible for transferring sialic acid from host glycoconjugates to mucins, also present on the parasite surface. The existence of several copies of TS genes has impaired the use of reverse genetics to study this highly polymorphic gene family. Using CRISPR-Cas9, we generated aTS knockout cell lines displaying undetectable levels of TS activity, as shown by sialylation assays and labeling with antibodies that recognize sialic acid-containing mucins. *In vitro* infection assays showed that disruption of aTS genes does not affect the parasite’s capacity to invade cells or to escape from the parasitophorous vacuole but resulted in impaired differentiation of amastigotes into trypomastigotes and parasite egress from the cell. When inoculated into mice, aTS mutants were unable to establish infection even in the highly susceptible gamma interferon (IFN-γ) knockout mice. Mice immunized with aTS mutants were fully protected against a challenge infection with the virulent T. cruzi Y strain. Altogether, our results confirmed the role of aTS as a T. cruzi virulence factor and indicated that aTS play a major role during the late stages of intracellular development and parasite egress. Notably, mutants lacking TS activity are completely avirulent in animal models of infection and may be used as a live attenuated vaccine against Chagas disease.

## INTRODUCTION

Trypanosoma cruzi is the protozoan parasite that causes Chagas disease, a debilitating and often life-threatening disease that affects 6 to 7 million people worldwide. Endemic in many Latin America countries, Chagas disease has spread to places in Europe, Australia, and areas south of the United States due to increased international migration. Although the disease is curable if treatment is initiated soon after infection, during the chronic phase, in which about 30% of infected people develop cardiac or digestive alterations, drug treatment is poorly effective in most cases. [https://www.who.int/en/news-room/fact-sheets/detail/chagas-disease-(american-trypanosomiasis)]. Therefore, the search for better treatments as well as for a vaccine that can prevent Chagas disease remains a challenge that requires a deeper understanding of the parasite biology.

T. cruzi has a complex life cycle with three main forms that alternate between its invertebrate and vertebrate hosts. Once inside the mammalian cell, trypomastigotes differentiate into replicative amastigotes, which multiply in the host cell cytoplasm and differentiate again into motile, infective trypomastigotes that, once released, propagate the infection by invading other cells (1). One of the main characteristics of the infective trypomastigote is the presence of large families of surface proteins, among them trans-sialidase (TS), one of the most studied virulence factors of T. cruzi. Although the parasite is unable to synthesize sialic acid (SA), the surface of bloodstream trypomastigotes is covered by this molecule thanks to the ability of TS to transfer SA from host glycoconjugates to β-galactopyranosyl residues of mucins, another family of surface glycoproteins (2, 3). The most common SA, *N*-acetylneuraminic acid, is a negatively charged nine-carbon sugar that is widely present in mammal tissues, being involved in different biological processes (4). Various pathogens have evolved to use SA beneficially as a nutrient, to mediate binding to host cells, or to coat themselves to evade host immune responses (4). Several studies indicated that during T. cruzi infection, SA contributes to parasite survival both in the insect vector and in the mammalian host (5).

Trans-sialidases are encoded by the largest gene family present in the T. cruzi genome (6) with its more than 1,000 genes classified into 8 different groups based on the presence of key TS motifs (7). Only members from group I have catalytic activity. Active TS (aTS) can be identified by the presence of a conserved tyrosine (Tyr^342^) positioned in the active-site floor and involved in the formation of a covalent glucosyl-enzyme intermediate (7–10). Very similar proteins which do not show transferase or hydrolytic activity due to a naturally occurring mutation that changes Tyr^342^ to a histidine (8) were named inactive TS (iTS). Active TS also has a highly immunogenic 12-amino-acid tandem repeats (DSSAHS/GTPSTPV/A) at the C-terminal portion, known as shed-acute-phase-antigen (SAPA) repeats, with variable repeat numbers (11). A TS role during invasion of different cell types has been proposed since the first studies on this enzyme (12). The large majority of other genes from TS family encode glycoproteins without trans-sialidase activity. There are more than 1,000 copies encoding these glycoproteins, and it has been proposed that this highly polymorphic protein family has other crucial functions, such as to act as lectins and to display a variable repertoire of surface epitopes that contribute to immune evasion (13).

Evidence from various studies suggests that TS acts as a major T. cruzi virulence factor. Once in the cytoplasm, the parasite activates signaling pathways that control cell growth and survival through interactions between TS and Akt (14). During the initial phases of infection in the mammalian host, TS affects the innate immune response in many ways, including delaying the formation of inhibitory or neutralizing antibodies due to a strong response against SAPA repeats (11) and suppressing the production of interleukin 12 (IL-12) by dendritic cells (15). Although a strong protective CD8^+^ response is trigged by epitopes present on TS (16), it has been suggested that this response is counteracted by the induction of apoptosis in the thymus (17) and changes in sialylation of CD8^+^ T cells caused by TS (18).

In spite of all efforts to investigate the multiple roles played by TS during T. cruzi infection, many gaps remain in our understanding of TS activities and its importance for the establishment of the complex equilibrium between host and parasite. The large number of copies of aTS and iTS genes dispersed in the T. cruzi genome (6) has prevented the use of reverse genetics to address these questions. The advent of the CRISPR-Cas9 technology is now allowing gene function studies that focus on the large T. cruzi gene families (19). We reanalyzed the complete repertoire of aTS genes present in the T. cruzi CL Brener genome and used CRISPR-Cas9 to create mutant parasites in which most, if not all, aTS genes were disrupted. The aTS mutant cell lines were able to invade mammalian cells and escape from the parasitophorous vacuole as well as wild-type parasites but were unable to differentiate from amastigotes to trypomastigotes and to egress from host cells. *In vivo* infection showed that the highly attenuated mutant cell lines were still able to elicit a robust antibody and T-cell immune response and to protect mice against a challenge with a highly virulent T. cruzi strain.

## RESULTS

### The complete aTS gene repertoire and expression profile.

Based on the CL Brener cloned strain genome assembly version 52, which is available at www.TriTrypDB.org, 11 sequences belonging to TS group I have been identified as encoding aTS (7). Because CL Brener is a hybrid strain with a high repeat content, it is well known that there are inaccuracies in the assembly of its genome and in the estimation of gene copy numbers, particularly in regions containing multigene families. To better estimate the complete repertoire of aTS genes in CL Brener, we used sequencing data generated with the PacBio and Illumina platforms as well as Sanger reads used in the original CL Brener genome assembly. Long reads are essential to assemble regions with tandems of repeats, whereas the high coverage and base call accuracy obtained with Illumina allows correction of sequencing errors. Based on the presence of amino acid residues crucial for TS activity, such as the conserved Asp box, the VTVxNVxLYNR motif, and Tyr^342^, we identified a total of 16 sequences that potentially encode aTS in the CL Brener genome (see Table S1 and Fig. S1 at https://figshare.com/s/9737443c64137bb5ab88). Three of these sequences contain premature stop codons and likely correspond to pseudogenes. As shown in Table S1, eight sequences identified in the new assembly using PacBio have more than 99% identity with sequences present in the original CL Brener assembly available at TriTrypDB, whereas eight other sequences showed a level of divergence that prevents a precise assignment to a specific sequence in TriTrypDB. Four sequences encoding aTS contain SAPA repeats at their C termini, with repeat content that varies from 4 to 29 repeats. One sequence containing 12 SAPA repeats corresponds to a pseudogene. Besides these five sequences, only one more sequence encoding an inactive TS (not shown in Table S1, also has a SAPA domain at its C terminus (Fig. S2 at https://figshare.com/s/9737443c64137bb5ab88). All but one aTS sequence have a glycosylphosphatidylinositol (GPI) anchor signal. Based on these analyses and considering a total of about 1,400 copies of TS sequences in the CL Brener diploid genome (6), it can be estimated that about 1% of the TS gene family repertoire of CL Brener encodes active enzymes.

**FIG 1:**
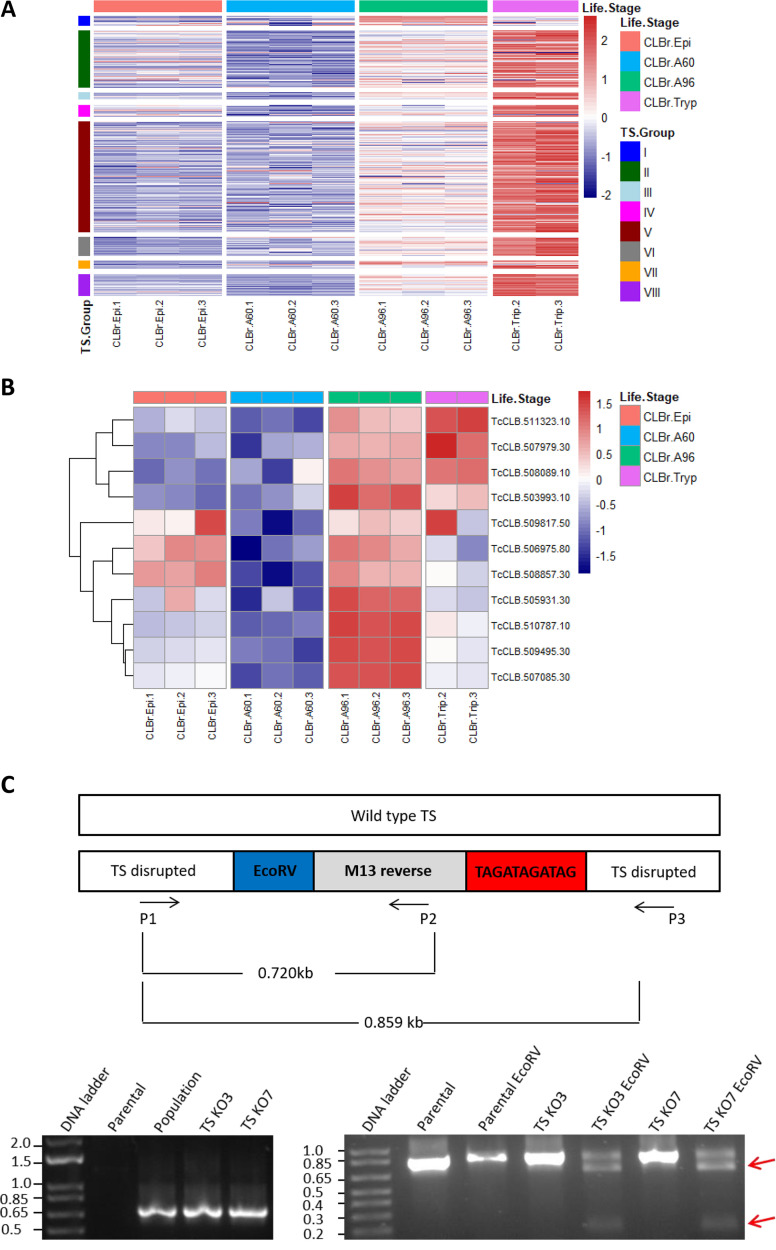
Expression profile of TS and analyses of parasites with disrupted aTS genes. (A and B) Heat maps representing changes in the expression levels in different stages of the T. cruzi life cycle (CL Brener strain) of 505 genes encoding TS belonging to all groups (A) and 11 aTS sequences shown in Table S1 (https://figshare.com/s/9737443c64137bb5ab88) (B). Rows represent individual genes, and columns represent RNA samples. The expression values are represented in a color grade scale, where red represents upregulation and blue represents downregulation. The genes were clustered according to the expression values across all genes. The different parasite stages and the different TS groups are represented in different colors on the *x* and *y* axes, respectively. (C) Genomic DNA from WT and transfected parasites were extracted and used as the template in PCRs with primers annealing in aTS coding sequences and the M13 sequence present in the donor template shown in the scheme above the agarose gel electrophoresis results. (Left) PCR products generated from DNA extracted from the parental cell line, the transfected population, and two cloned transfected cell lines (TS KO3 and TS KO7) using primers P1 and P2 and separated by agarose gel electrophoresis; (right) PCR products generated from DNA extracted from TS KO3 and TS KO7, using primers P1 and P3, before and after digestion with EcoRV restriction enzyme. Arrows indicate the digested products. DNA molecular size markers are in kilobases.

The expression profile of aTS genes was evaluated based on the previously described transcriptome sequencing (RNA-seq) data obtained from tissue culture trypomastigotes and intracellular amastigotes harvested at 60 and 96 h postinfection (hpi) of human fibroblasts (20). We also incorporated in this analysis RNA-seq data obtained from *in vitro* cultured epimastigotes of the CL Brener cloned strain, which were more recently reported (21). Since the RNA-seq reads were mapped to the T. cruzi CL Brener reference genome, available in TriTrypDB, TS mRNA levels based on RNA-seq data were evaluated for only 11 aTS genes. As shown in Fig. 1A, the RNA-seq analysis corroborates previous studies from many groups, including ours (7, 20), that showed increased expression of TS genes in trypomastigotes compared to epimastigotes and amastigotes. However, different from most TS genes, which encode mainly iTS, the majority of aTS genes have increased expression in amastigotes at 96 hpi compared to the other forms (Fig. 1B). Except for three aTS sequences that showed slightly increased expression in trypomastigotes (TcCLB.511323.10, TcCLB.507979.30, and TcCLB.508089.10) compared to amastigotes at 96 hpi and two aTS sequences presenting similar expression levels in epimastigotes and amastigotes (TcCLB.506975.80 and TcCLB.508857.30), all aTS sequences have increased expression in amastigotes at 96 hpi compared to the other parasite forms (Fig. 1B). Expression of all aTS is downregulated in replicative amastigotes, i.e., in amastigotes at 60 hpi. Therefore, the transcriptome data indicate that expression of aTS genes is upregulated during the final steps of the intracellular development, i.e., during differentiation of intracellular amastigotes into trypomastigotes. Since three aTS sequences that are upregulated in amastigotes at 96 hpi contain SAPA repeats, the RNA-seq data also indicate that the parasite exposes TS with SAPA repeats before it egresses from the cell.

### Targeting aTS and generation of aTS mutant cell lines.

A region containing 210 nucleotides spanning the conserved Tyr^342^ was selected to design two sgRNAs that target all aTS sequences (see Fig. S3 at https://figshare.com/s/9737443c64137bb5ab88). To generate the knockout cell lines, epimastigotes constitutively expressing green fluorescent protein (GFP)-tagged Cas9 were transfected with the two single guide RNAs (sgRNAs) together with an oligonucleotide to be used as a repair template during homologous recombination repair, as previously described (22). The repair template is a single-stranded oligonucleotide containing the EcoRV restriction site, the M13 reverse primer and three stop codons flanked by 25 nucleotides complementary to aTS sequences (see Fig. S3 at https://figshare.com/s/9737443c64137bb5ab88). After three rounds of transfection with sgRNAs plus oligonucleotide, parasites were cloned by serial dilution and total DNA was extracted. To screen for clones with disrupted aTS, DNA from various clones was used in PCRs with a forward primer annealing in the coding sequence of aTS sequences and a reverse primer complementary to the M13 sequence present in the repair template (Fig. 1C, left; primers P1 and P2). Amplification of fragments with the expected sizes (0.7 kb) showed the presence of parasites with disrupted aTS genes in the transfected population. Among a total of 16 clones analyzed, PCR analyses showed that 69% have the repair template inserted in an aTS gene disrupting its coding region. To estimate the proportion of aTS sequences that were disrupted in each transfected clone, DNA from two clones, named TS KO3 and TS KO7, were PCR amplified with two primers annealing in the coding sequence of aTS genes, and the PCR products were digested with EcoRV. As shown in Fig. 1C (right), EcoRV digestion of the 0.76-kb PCR product generated with primers P1 and P3 resulted in two fragments of 0.60 kb and 0.16 kb. It is noteworthy that not all PCR products were digested, indicating that in both clones, recombination with the donor oligonucleotide containing stop codons and the EcoRV restriction site would not have occurred within all copies of the aTS gene family. Alternatively, primers P1 and P3 amplified iTS sequences. To further estimate the proportion of aTS sequences disrupted and confirm the insertion of the donor oligonucleotide, amplicons generated with DNA from the TS KO7 cell line and primers P1 and P3 were cloned into pCR2.1-TOPO TA vector. PCR screening performed with individual colonies after E. coli transformation using M13 forward and reverse primers showed amplification products with one or two distinct sizes (1.1 kb and 0.8 kb). The presence of two PCR bands identified plasmid clones containing a disrupted aTS sequence, since two annealing sites for the M13 reverse primer are present in pCR2.1-TOPO plasmids containing aTS sequences that recombined with the oligonucleotide repair template (see Fig. S4 at https://figshare.com/s/9737443c64137bb5ab88). The PCR screening with M13 primers indicated that in the TS KO7 mutant cell line, about 55% of sequences amplified with primers P1 and P3 were disrupted. Sequencing of pCR2.1-TOPO plasmids extracted from six colonies that generated two bands after PCR with M13 primers confirmed the integration of the repair template and the disruption of aTS coding sequences (see Fig. S4 at https://figshare.com/s/9737443c64137bb5ab88).

To ascertain the accuracy of our strategy in targeting all aTS genes and evaluate the potential occurrence of off target alterations as well as genomic rearrangements in mutant parasites, we performed whole-genome sequencing of parasites expressing Cas9 and TS KO7 mutant parasites, using the Illumina sequencing platform. A total of ∼3 Gbp of genomic sequences was generated for each of the two libraries. In addition, we also analyzed Illumina reads from a CL Brener wild-type (WT) isolate which has been maintained in culture in our lab. All libraries were mapped on the CL Brener reference genome, obtained from TriTrypDB (v52). Based on the analysis of discordant alignments of paired-end reads, we did not find evidence for the occurrence of major genomic rearrangements either in the Cas9 parental cell line or in the TS KO7 mutant (see Table S2 at https://figshare.com/s/9737443c64137bb5ab88). Compared with the genome of the CL Brener WT isolate, the percentage of discordant paired ends is similar to the values obtained with the Cas9 cell line and TS KO7 mutant. Although we cannot exclude the possibility that misassembly of the reference CL Brener genome may have inflated these numbers for all three isolates, these analyses indicated that no major genomic rearrangement events occurred in parasites expressing Cas9 or TS KO7 mutants beyond the expected levels of genetic variability that may occur when T. cruzi parasites are cultivated for long periods. To further investigate the accuracy of our strategy of targeting aTS genes and the potential occurrence of off-target alterations, reads from both Cas9 and TS KO7 genomic libraries were searched for a 27-nucleotide (nt) sequence that corresponds to a fragment of the donor oligonucleotide and is absent from the reference CL Brener genome. As expected, only the TS KO7 library contained this sequence, totalizing 52 reads. When mapped on the CL Brener v52 genome, all 52 reads were found to be part of TS genes. At least one of the 52 donor-containing reads mapped on each one of the aTS targeted for disruption shown in Table S1 (https://figshare.com/s/9737443c64137bb5ab88). These reads also mapped to five other aTS genes (TcCLB.507611.260, TcCLB.506895.80, TcCLB.508859.118, TcCLB.506923.10, and TcCLB.508501.320) but not to any other T. cruzi gene or genomic region. Thus, based on whole-genome analyses, we were able to confirm that the alterations caused by homologous recombination repair of the double-strand break generated in the Cas9-expressing parasites after transfection with the sgRNA and the oligonucleotide donor occurred mainly on the target aTS genes.

### Molecular characterization of aTS mutant cell lines.

**FIG 2:**
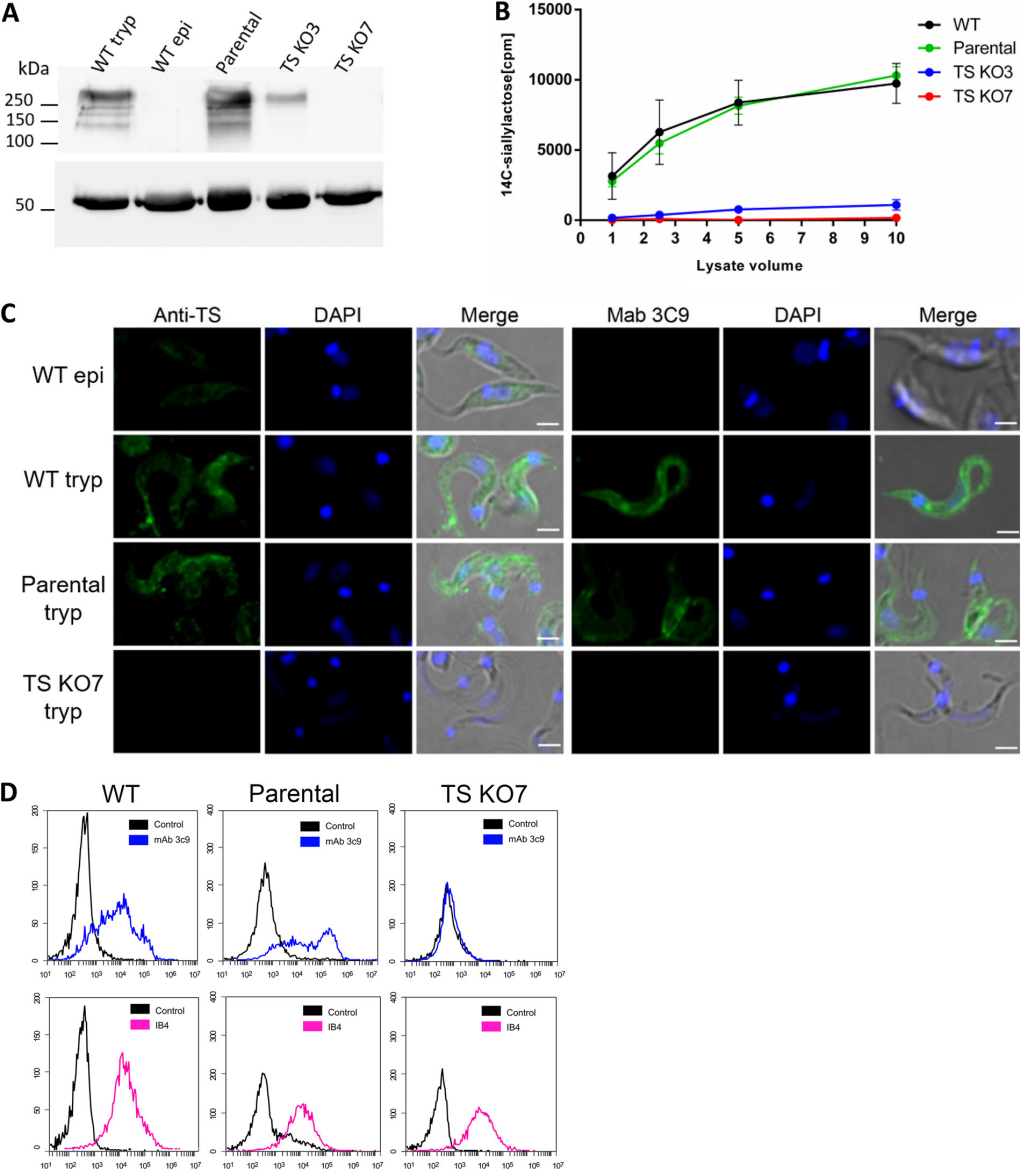
TS expression and activity in WT and aTS mutants. (A) Total protein extracts from WT trypomastigotes (WT tryp), WT epimastigotes (WT epi), trypomastigotes of parental cell line (parental) and trypomastigotes of TS KO3 and TS KO7 were separated in 7.5% polyacrylamide gels, transferred to nitrocellulose membranes, and incubated with anti-TS antibody. The bottom panel shows incubation of the membrane with anti-tubulin antibody as a loading control. (B) TS activity assays using [^14^C]siallyllactose and cell lysates from WT trypomastigotes, trypomastigotes of the parental cell line, and trypomastigotes of TS KO3 and TS KO7. (C) WT trypomastigotes or epimastigotes and trypomastigotes of the mutant cell line TS KO7 were fixed with 4% paraformaldehyde and incubated with anti-TS antibody (left) or with 3C9 antibody (right), following incubation with Alexa-labeled secondary antibody. Bars, 2 μm. (D) WT trypomastigotes or trypomastigotes of the mutant cell line TS KO7 were fixed with 4% paraformaldehyde and incubated with 3C9 monoclonal antibody (top) or with biotinylated IB4 isolectin (bottom) followed by incubation with Alexa-labeled anti-mouse secondary antibody or streptavidin phycoerythrin. Control samples correspond to parasites incubated with PBS.

To evaluate the expression of aTS genes in the mutant cell lines, Western blot analyses were performed with total protein extracts from epimastigotes and trypomastigotes from WT parasites and the two mutant cell lines using anti-TS antibodies. This polyclonal antibody was raised in mice against a recombinant aTS that contains 19 SAPA repeats. When tested against the full-length recombinant protein, the protein containing only the N-terminal catalytic domain, and the protein containing only the C-terminal SAPA repeats, these antibodies strongly recognized SAPA repeats, which are present in aTS (see Fig. S5 at https://figshare.com/s/9737443c64137bb5ab88). As shown in Fig. 2A, TS containing SAPA repeats are present in protein extracts from WT trypomastigotes as well as in trypomastigotes from the parental cell line (parasites expressing Cas9). As expected, TS containing SAPA repeats were absent in epimastigote extracts (23). Whereas only faint bands were detected in protein extracts from trypomastigotes of the clone TS KO3, no detectable reaction with the anti-TS-SAPA antibodies was observed in protein extracts from trypomastigotes of the clone TS KO7. Consistent with the Western blot results, TS activity assays using different amounts of parasite lysate demonstrated that TS activity was also significantly reduced in clone TS KO3 and was completely abolished in the TS KO7 cloned cell line (Fig. 2B). In contrast, and also consistent with the Western blot, cell lysates from the parental cell line showed a level of TS activity similar to that in WT parasites. Loss of TS expression at the parasite surface as well as TS activity in the mutant cell line TS KO7 was confirmed by immunofluorescence analyses using anti-TS-SAPA antibody as well as with the 3C9 monoclonal antibody, which recognizes an epitope in sialylated mucins (24, 25). Figure 2C shows that, unlike epimastigotes of the WT CL Brener clone, trypomastigotes from WT cells as well as from the parental cell line express TS containing SAPA repeats and sialylated mucins on their surface. In contrast, no TS labeling or labeling with monoclonal antibody (MAb) 3C9 was observed on the surface of trypomastigotes from the mutant cell line TS KO7. Flow cytometry analyses also showed that, in contrast with labeling with 3C9 MAb, no difference was observed in the binding of the isolectin IB4 from Bandeiraea (Griffonia) simplicifolia (Sigma-Aldrich), which has a high affinity for nonreducing α-Gal residues. The similar labeling with IB4 observed in parental trypomastigotes and trypomastigotes from TS KO7 indicates that α-Gal epitopes were not altered in the aTS mutants (Fig. 2D, bottom panels). Also, the lack of SA at the surface of trypomastigotes in mutant cell line TS KO7 did not affect its resistance to lysis by human complement (data not shown).

### Evaluation of *in vitro* and *in vivo* infection capacity of aTS mutant cell lines.

**FIG 3:**
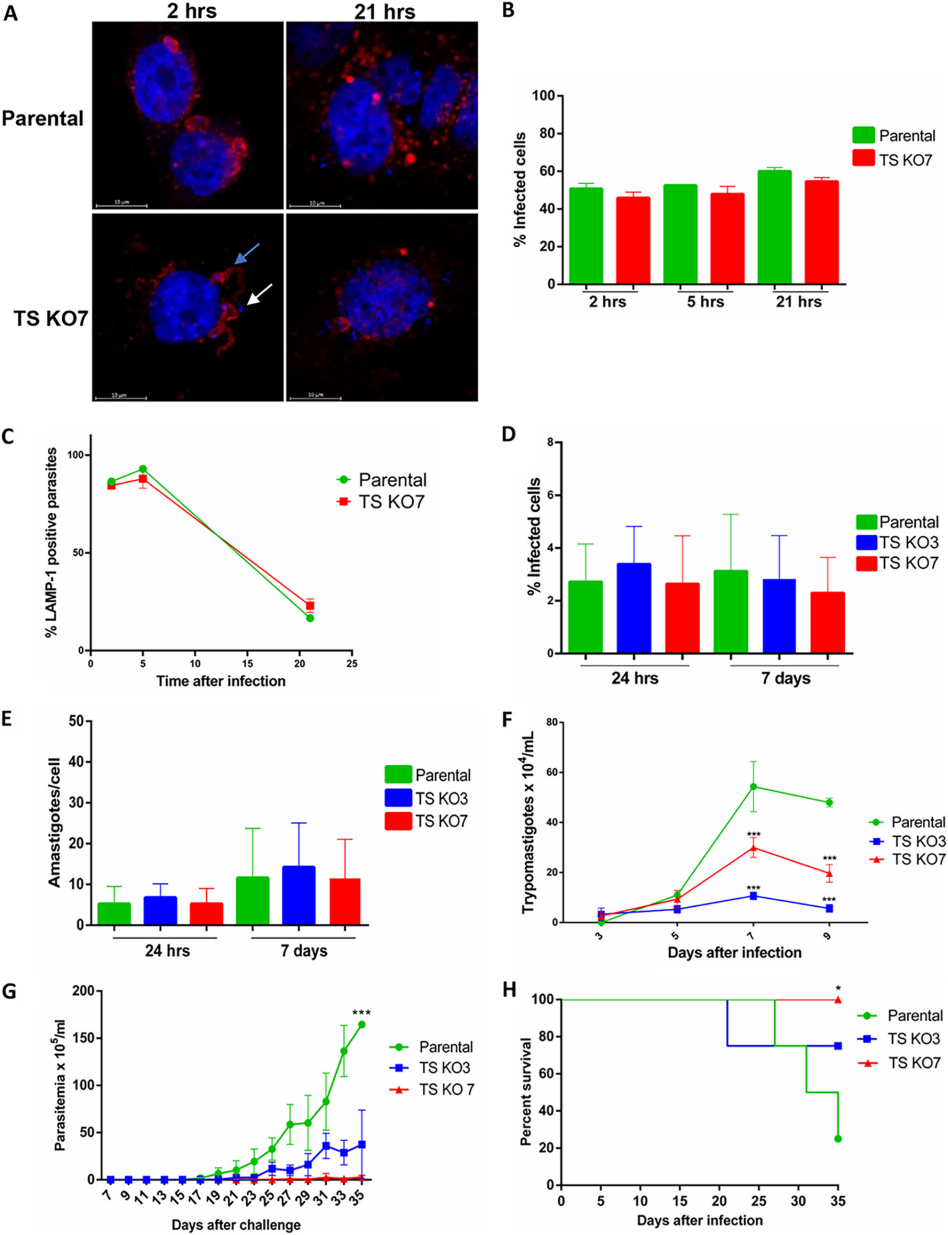
Infection capacity of aTS KO mutants. HeLa cells were infected with trypomastigotes from the parental cell line or the mutant cell line TS KO7 for 1 h, washed with PBS, and fixed with paraformaldehyde 2, 5, and 21 h after infection. (A) Fixed cells were incubated with anti-LAMP-1 (MAb H4A4) followed by anti-mouse–Alexa Fluor 555. After nucleus staining with DAPI, images were acquired with a 63× objective in the Zeiss LSM 880 Airyscan confocal microscope. Arrows show parasites that colocalized with LAMP-1 (blue arrow) and parasites that are in the cytosol and did not colocalize with the lysosomal marker (white arrow). (B and C) Percentages of infected cells (B) and percentages of parasites that colocalized with LAMP-1 (C) at 2, 5, and 21 h postinfection. Monolayers of LLC-MK2 cells were infected with trypomastigotes from the parental cell line or the mutant cell lines TS KO3 and TS KO7. (D and E) Twenty-four hours or 7 days after infection, the percentages of infected cells (D) and the numbers of amastigotes in infected cells (E) were determined. Statistical analyses were performed with one-way analysis of variance (ANOVA) with Tukey’s multiple comparisons as a posttest. No significant statical difference was found in the percentage of infected cells or in the number of amastigotes between cells infected with the parental cell line and those with the two mutant cell lines. (F) The numbers of trypomastigotes released in the supernatant were also determined up to 9 days after infection of LLC-MK2 cells with trypomastigotes from the parental cell line and the two mutant cell lines. Statistical analysis was performed with two-way ANOVA, with Bonferroni correction for multiple testing (****, *P* < 0.0001). (G and H) IFN-γ KO mice were infected with trypomastigotes of the parental cell line or the mutant cell lines TS KO3 or TS KO7, and parasitemia (G) and survival rates (H) were followed for 35 days. Statistical analysis for parasitemia was performed with two-way ANOVA with Tukey’s multiple comparisons as a posttest (***, *P* < 0.0001).

Because members of the TS family have been described as parasite factors involved in cell invasion, we asked whether the aTS mutants have a reduced invasion capacity or reduced capacity to escape from the parasitophorous vacuole (PV) that is formed immediately after parasite invasion. HeLa cell monolayers were incubated for 1 h with culture-derived trypomastigotes at a multiplicity of infection (MOI) of 100, and the percentages of infected cells were evaluated 2, 5, and 21 h after infection. Since no differences in the percentages of cells infected were observed 2 h postinfection (Fig. 3A and B), we concluded that the absence of aTS does not affect invasion. In addition, no differences in the numbers of parasites that colocalize with the lysosomal marker LAMP-1, a marker for the recently formed PV, were observed when the parental cell line and the TS KO7 mutant were compared at all time points analyzed (Fig. 3C). The *in vitro* infection capacity of the two aTS knockout (KO) cell lines, TS KO7 and TS KO3, were also evaluated using other cell lines such as LLC-MK2 cells (Fig. 3D to F) or the THP-1 monocytic cell line (data not shown). In these experiments, cell monolayers were incubated at an MOI of 10 trypomastigotes/cell for 3 h, and the percentage of infected cells (Fig. 3D), the numbers of intracellular amastigotes per cell (Fig. 3E), and the numbers of trypomastigotes released in the culture supernatant (Fig. 3F) were evaluated. Again, no differences in the percentages of cells infected or in the numbers of amastigotes per cell were observed with the mutant cell lines TS KO3 or TS KO7 compared with the parental Cas9 cell line. In contrast, significant differences in the numbers of trypomastigotes released in the supernatant were detected when cells infected with both mutant cell lines were compared with cells infected with the parental cell line: on day 9 postinfection, cells infected with TS KO3 and TS KO7 released 50 to 70% fewer trypomastigotes than cells infected with the parental cell line (Fig. 3F). No statistically significant difference was observed between the numbers of trypomastigotes released in the culture supernatant from cells infected with TS KO3 and those with TS KO7.

Taken together, these results indicate that the lack of or reduced TS activity does not affect parasite invasion, its escape from the PV into cytoplasm, or the amastigote replication capacity. In contrast, the absence of TS activity negatively affects amastigote/trypomastigote differentiation and the release of trypomastigotes from infected cells. The presence of rounded amastigote-like forms in culture supernatants from cells infected with mutant cell lines and the presence for longer times of amastigotes inside cells infected with TS mutants than in cells infected with the parental cell line were noteworthy. These observations further indicate that amastigotes from mutant cells lines have an impaired capacity to differentiate into trypomastigotes. It is also possible that rupture of heavily infected cells before differentiation of amastigotes into trypomastigotes may cause the release of amastigotes into the supernatant.

To evaluate the *in vivo* infection capacity of the TS mutant cell lines, 10,000 tissue culture-derived trypomastigotes of the parental or the mutant cell lines were inoculated into BALB/c mice and the parasitemia was followed for 35 days. Since no parasites were detected in the bloodstream of BALB/c mice inoculated either with the parental cell line or with the aTS mutants, we used gamma interferon (IFN-γ) KO mice to perform the *in vivo* infection analyses. It is well known that prolonged *in vitro* cultivation results in the selection of attenuated parasites with low infectivity in immunocompetent animals (26). It is also known that IFN-γ is a determinant cytokine for parasite control and that T. cruzi infection of IFN-γ KO mice results in high parasitemia and mortality rates (27). In contrast to infection with the parental cell line, which showed a drastic increase in parasitemia 20 days postinfection and 80% mortality on day 35, IFN-γ KO mice infected with the TS KO7 mutant cell line displayed no parasitemia and showed 100% survival 35 days postinfection (Fig. 3G and H). It is noteworthy that IFN-γ KO mice infected with TS KO3, which has reduced TS activity, also had low parasitemia and increased survival (80%) compared to mice infected with the parental cell line (Fig. 3G and H).

### Immunization of BALB/c mice with aTS mutants and protection against a challenge with a virulent T. cruzi strain.

**FIG 4:**
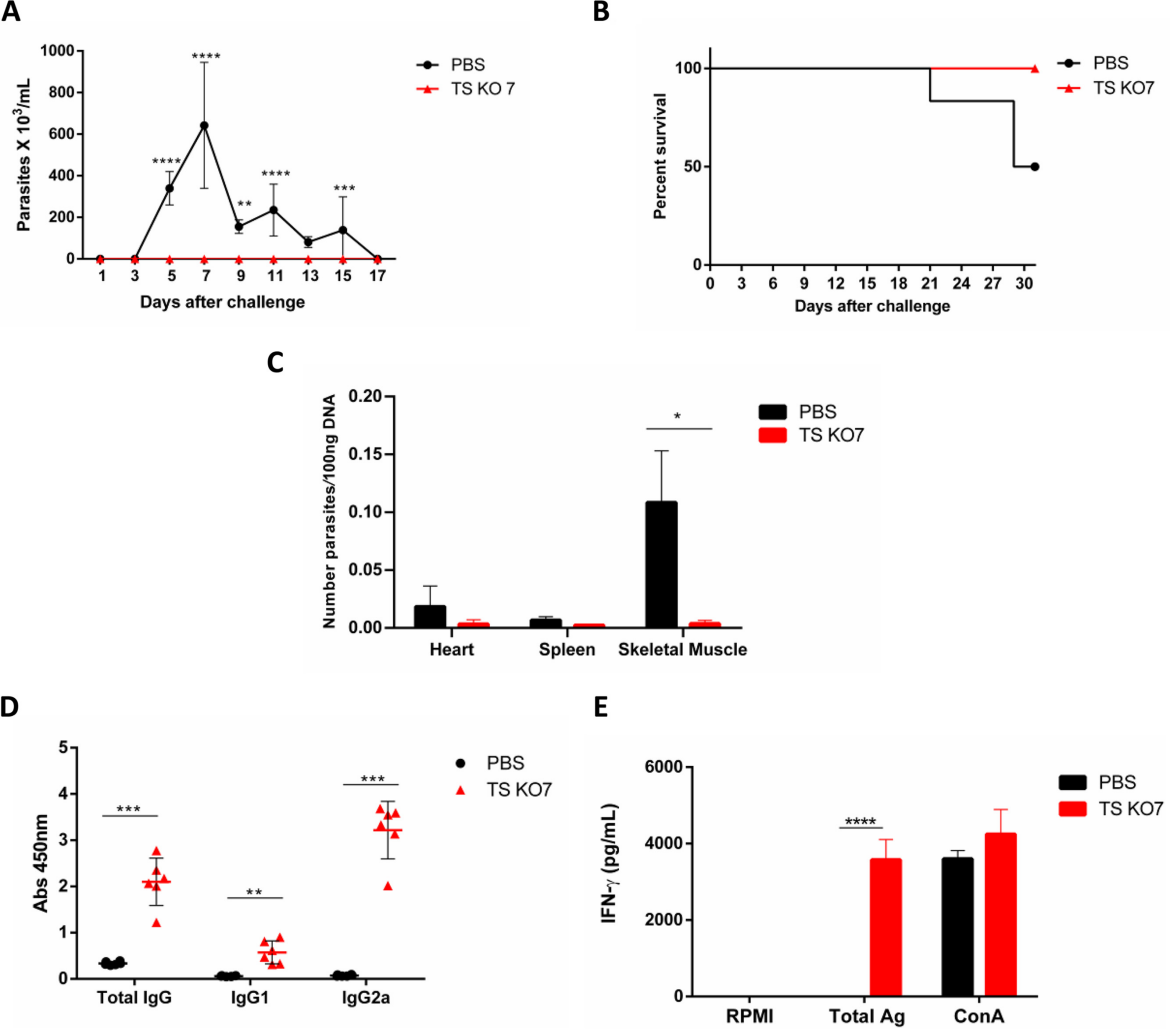
Protection capacity of aTS KO mutants. (A to C) BALB/c mice were inoculated with trypomastigotes of the mutant cell line TS KO7 or with PBS, and after 30 days were challenged with trypomastigotes from strain Y. Parasitemia (A) and survival (B) were determined. Statistical analysis of parasitemia was performed with two-way ANOVA with Sidak’s multiple comparisons as a posttest (****, *P* < 0.0001). Thirty days after challenge infection, mice were sacrificed, mouse hearts, spleen, and skeletal muscle were removed, DNA was extracted, and the relative amount of parasite DNA in each tissue was determined by real-time PCR (C). Statistical analysis was performed with a multiple *t* test (*, *P* < 0.01). (D and E) Twenty days after being inoculated with trypomastigotes of the mutant cell line TS KO7 or with PBS, mice were sacrificed and serum was collected for determination of levels of total IgG, IgG1, and IgG2a (D). Statistical analysis was performed with a multiple *t* test (***, *P* < 0.0001; **, *P* < 0.001). On day 20, mouse spleens were also removed, and IFN-γ was quantified in the supernatant of spleen cell cultures stimulated with WT trypomastigote antigen extracts or with concanavalin A (E). Statistical analysis was performed with two-way ANOVA with Sidak’s multiple comparisons as a posttest (****, *P* < 0.0001).

Infection experiments with the highly susceptible IFN-γ KO mice indicated that the aTS mutant cell line TS KO7 has characteristics of a fully attenuated parasite. To verify whether the T. cruzi aTS-null mutant can be used in immunization protocols to protect mice against a challenge infection, we inoculated one group of BALB/c mice with 5,000 trypomastigotes from the TS KO7 cell line and a second group with phosphate-buffered saline (PBS). Thirty days after, both groups of mice were challenged with 10,000 trypomastigotes of the highly virulent Y strain of T. cruzi. As shown in Fig. 4A, we did not detect parasites in the bloodstream of mice inoculated with TS KO7 mutant cell line, indicating that these animals were fully protected against the challenge with the Y strain. We cannot exclude the possibility that parasitemia of mice inoculated with TS KO7 was below the limit of detection of the method, which is about 10^4^ parasites/mL (28). In contrast, control mice that received PBS showed high levels of parasitemia and mortality rates (Fig. 4A and B). Quantitative PCR analyses with DNA extracted from various mouse tissues also showed no detectable T. cruzi DNA in heart, spleen, or muscle tissues from mice that were immunized with TS KO7 prior to challenge, in contrast with control mice in which high levels of parasite DNA was detected in the skeletal muscle (Fig. 4C). To investigate the humoral and immune responses in immunized mice, sera as well as spleens from these animals were collected 30 days after inoculation with the TS KO7 mutant, before the challenge with the virulent strain. As shown in Fig. 4D and E, immunized mice displayed high levels of IgG2a in the serum as well as high IFN-γ production by splenocytes after stimulation with whole-parasite extracts. These results indicated that inoculation with the TS KO7 mutant is an effective way to induce a protective Th1 response that is able to control a subsequent infection with a virulent parasite strain. Taken together, the data presented here not only confirm that the aTS mutants are unable to cause infection in mice, further demonstrating that aTS acts as an important parasite virulence factor, but also show that an attenuated strain with disrupted aTS genes may be used as a vaccine that can induce protection against T. cruzi infection.

## DISCUSSION

Thirty-five years ago, it was demonstrated that T. cruzi can enzymatically transfer SA to its surface (29) and that a trans-sialidase activity was detected in trypomastigotes (30). Over the years, many studies have provided evidence in support of TS as an important T. cruzi virulence factor (13, 31). However, due the multiple gene copies and the lack of RNAi machinery in T. cruzi, our capacity to generate TS knockout parasites was limited, and so were the studies addressing the primary role of this enzyme on parasite-host cell interaction. Here, we characterized two parasite mutant cell lines, one with significantly reduced TS activity and another with undetectable TS activity, that were generated by using CRISPR-Cas9 technology. While earlier studies have suggested that TS is a key element for invasion (12) and parasite escape from the parasitophorous vacuole (32), we found that aTS knockout parasites invade and replicate in the cytoplasm at rates similar to those of WT parasites, but the absence of TS activity causes a severe impairment of the parasite egress from the host cell.

TS are glycosylphosphatidylinositol (GPI)-anchored surface proteins that may be shed into the bloodstream of the mammalian host within microvesicles or by the action of a parasite phosphatidylinositol-specific phospholipase C (5, 33). A careful examination of the complete genome of the CL Brener reference strain showed the presence of 16 aTS sequences, which corresponds to about 1% of the full repertoire of TS genes. Our estimation of such a small proportion of aTS genes is consistent with studies based on quantitative PCR analyses determining that the genomes of various T. cruzi isolates harbors between 28 to 32 copies of aTS-coding sequences (34). As has been shown by extensive reviews (13, 31), studies performed by different groups have proposed multiple roles for this enzyme, including parasite attachment to and detachment from insect gut endothelia, invasion of mammalian cells, subversion of cell signaling machinery, evasion of the humoral response, and disruption of an effective cellular response against the parasite. Because we were able to disrupt most parasite genes encoding active forms of the enzyme, while maintaining intact inactive members of the family, we are now able to investigate the impact of preventing the transfer of SA during *in vitro* and *in vivo* infection.

In earlier studies, we characterized a naturally attenuated isolate (clone CL-14), which does not cause infection in immunocompetent mice but induces strong protective immunity (35, 36). Comparative transcriptome analyses and immunoblots showed a largely decreased expression of TS genes in the CL-14 clone (20) compared to the virulent CL-Brener clone. Our previous transcriptome studies comparing CL Brener and CL-14 are also consistent with the expression profiling of genes encoding aTS, which are upregulated during the transition of intracellular amastigotes at 60 hpi to amastigotes at 96 hpi. Similar to the results obtained with the aTS mutants, *in vitro* infection assays showed that the infection capacity of CL-14 trypomastigotes was not different from that of CL Brener, whereas the numbers of trypomastigote released from cells infected with CL-14 were significantly lower than the numbers in cells infected with CL Brener (20). However, these differences could not be attributed solely to the diminished expression of aTS, since other differences, not yet identified in the CL Brener and CL-14 genomes, could also contribute to their distinct virulent phenotypes. Distinct from the comparison between CL Brener and CL-14 cloned isolates, whole-genome comparisons between the TS KO7 mutant and the parental CL Brener strain that has been modified only to express Cas9 did not reveal any other major differences besides the presence of the donor sequence in the aTS mutant, which introduced stop codons in aTS genes. RNA-seq analyses will help provide further evidence regarding the absence of other differences besides the disruption of aTS genes between the WT, the parental cell line, and the aTS mutants.

Once we generated CL Brener cell lines with disrupted aTS genes, we were able to establish a correlation between TS expression/activity and infection capacity both *in vitro* and *in vivo*. Intriguingly, *in vitro* infection assays with two mutant cell lines showed that TS activity is not essential for invasion, for establishing infection, or for escape of the parasite from the PV. Hence, our results are consistent with several other studies that indicate a role of inactive members of the TS family, through their lectin binding domain, in the process of adhesion and/or internalization of trypomastigotes into host cells (13, 37, 38). The results of *in vitro* infection assays in which colocalization of intracellular parasites with LAMP-1-positive vacuoles was determined did not confirm previous observations suggesting a role for TS in parasite escape from PV. This may be due to the fact that, in these previous studies, infection was done with a different parasite strain and with metacyclic trypomastigotes, instead of culture-derived trypomastigotes (TCTs) (32). Unexpectedly, our data also show that disruption of aTS genes has a major impact on the final steps of parasite intracellular development when amastigotes differentiate into trypomastigotes and the parasite egress from the host cell. Based on the studies of mechanisms of viral infection (39) and the findings described here, we propose a role similar to that of hemagglutinin (HA) and neuraminidase (N) during influenza virus entry and budding from the cell. By binding to SA, the HA mediates invasion, and by cleaving SA bound to HA, N releases nascent virus particles from infected cell (40). In T. cruzi, these HA and N functions are unified in a single TS molecule (33). While the TS lectin activity binds to SA on the host cell surface and mediates invasion, the enzymatic activity of TS cleaves SA and mediates T. cruzi egress from the host cell.

With the aim of developing preventive or prophylactic vaccines against Chagas disease, several groups have tried different vaccine formulations, including live attenuated vaccines (41), vaccines using killed parasites (42), recombinant proteins (43), DNA vaccines (44), and modified adenoviruses expressing T. cruzi proteins (45). However, the immunity is rarely complete, in contrast to attenuated T. cruzi parasites such as the CL-14 clone (36). Since TS KO7 mutants were able to infect cells but have impaired capacity to release trypomastigotes from the infected cell, propagation of the infection cannot occur in the animal. It is noteworthy that a correlation between levels of TS activity in the mutants and parasitemia in the infected animals was observed. Thus, the complete absence of parasites in the bloodstream even in the highly susceptible IFN-γ KO mice may be explained by the fact that trypomastigotes lacking TS activity do not egress from the infected cell. At the same time, the high IFN-γ production by splenocytes from TS KO7-immunized BALB/c mice indicates that the low numbers of infected cells may be sufficient for the attenuated TS mutant to generate a strong cellular immune response, which resulted in resistance to a challenge with a virulent strain. Regardless, the results presented here provide a paradigm shift in our understanding in the role of aTS in parasite-host interaction and provide a first step toward the development of a novel vaccine strategy that is urgently needed to prevent this highly neglected disease.

## MATERIALS AND METHODS

### Sequence identification and expression analyses of trans-sialidases.

Sequences encoding aTS were retrieved from the scaffolds generated from Illumina HiSeq paired reads (mean read length, 100 bp) and reads obtained with the PacBio platform (read length of 100 to 110,848 bp) for the CL Brener strain. These scaffolds were subjected to BLAST analyses against a 210-nt sequence corresponding to the conserved catalytic domain indicated in Fig. S1(https://figshare.com/s/9737443c64137bb5ab88). All TS coding sequences that were selected after the BLAST search were also evaluated for the presence of Asp box and VTV motifs, signal peptide using Signal-P (46), GPI anchor with PredGPI (47), and as SAPA repeats. Global alignments of all aTS sequences were performed with MAFFT (48). The corresponding aTS genes were also retrieved from TriTrypDB v52 (http://TriTrypDB.org) after BLAST searches with each sequence used as a query. The RNA-seq data from T. cruzi CL Brener strain during the different life cycle stages, including epimastigotes, trypomastigotes, and amastigotes at 60 and 96 h postinfection, were obtained from our previously published data (20, 21). Raw counts were normalized to counts per million (cpm) and converted in log_2_ cpm. Data normalization and the differential expression analyses were performed using DESeq2. Reads that aligned to TS genes were selected, and the expression values were obtained by the subtraction of log_2_ cpm of each specific gene from the gene’s average across all samples and plotted as a heat map as described previously (20).

### Design and *in vitro* synthesis of single guide RNAs.

sgRNAs containing 20 nucleotides complementary to sequences present exclusively in aTS genes were designed using the Eukaryotic Pathogen CRISPR guide RNA/DNA design tool available at http://grna.ctegd.uga.edu/. The region near the codon that encodes Tyr^342^ was selected, and two sgRNAs with best scores were selected. The two sgRNAs targeting aTS were PCR amplified using a forward primer containing the aTS sequences and the T7 promoter sequence and a reverse primer that anneals with the sequence of Streptococcus pyogenes Cas9 scaffold of sgRNA, as described before (22). All primer sequences are available in Table S3 (https://figshare.com/s/9737443c64137bb5ab88). The resulting PCRs were purified using a PCR cleanup kit (Macherey-Nagel), and approximately 2 μg of PCR product was used in an *in vitro* transcription reaction following the manufacturer’s instructions (MEGAshortscript kit; Thermo Fisher). The reaction mixtures were incubated at 37°C for 16 h, and the RNA products were purified with phenol-chloroform as described elsewhere (22).

### Generation of aTS mutant cell lines, PCR amplification, restriction enzyme digestion, and sequence analyses of mutant cell lines.

A total of 4 × 10^7^ epimastigotes from a CL Brener clone constitutively expressing Cas9 from Streptococcus pyogenes (22) were transfected three times with both sgRNAs plus repair template using the Amaxa Nucleofector (Lonza) following previously described protocols (22). After transfection, parasites were cloned by serial dilution in 96-well plates. All procedures involving live, genetically modified T. cruzi were made in a biosafety level 2 facility and approved by the institutional biosecurity committee. DNA was extracted from cloned, transfected epimastigote cell lines with an Illustra blood genomicPrep Mini Spin kit (GE Healthcare). Clones with disrupted aTS gene sequences were identified by PCRs with primers aTSs F and M13 R. After PCRs, amplicons generated with primers P1 and P3 were incubated with EcoRV for 3 h, and the resulting products were separated by agarose gel electrophoresis. Cloning of PCR product derived from TS KO7 were performed using pCR 2.1-TOPO (Invitrogen) and PCR screening using primers M13 F and M13 R. Sequencing was performed with ABI 3730 Analyzer and the BigDye Terminator v3.1 cycle sequencing kit (Applied Biosciences).

### Whole-genome sequencing analysis of parasites overexpressing Cas9, the TS KO7 mutant, and WT parasites.

Whole-genome sequencing of parasites expressing Cas9 and TS KO7 parasites was performed using the Illumina platform (TruSeq DNA Nano 550-bp library, 300 PE [paired-end]). A total of ∼3 Gb of genomic sequences was generated for each of the two libraries. Illumina reads from a WT CL Brener isolate previously generated by our group (NCBI SRA accession number SRR6357354) was also used to evaluate the expected genomic variability in different parasite cultures. The read libraries’ base quality was evaluated with FastQC (https://www.bioinformatics.babraham.ac.uk/projects/fastqc/) and Trimmomatic v.0.39 (49) to remove adaptor sequences and select reads that had mean Phred quality scores higher than 20 and that were larger than 100 nucleotides. Polished reads from each of these three libraries were mapped separately on both haplotypes of CL Brener reference genome, Esmeraldo-like and non-Esmeraldo, obtained from TriTrypDB (v52; www.tritrypdb.org), using BWA-mem v.0.7.12 (50). SAMtools v1.1 (51) was used to manipulate the alignment files and to estimate the proportion of mapped reads. Analyses of genomic rearrangements were performed based on the percentage of discordant alignments of paired-end reads to the reference genome, using SAMtools flagstat. To identify the locations for the insertion of the exogenous donor sequence, reads from the Cas9 and TS KO7 genomic libraries were search for the exact sequence CGCCAGGGTTTTCCCAGTCACGACGAT using SeqKit (https://bioinf.shenwei.me/seqkit/). This 27-nt sequence corresponds to a fragment of the donor oligonucleotide and is absent from the reference CL Brener genome, which integrates into the genome after dsDNA repair by homologous recombination. Reads that contained this sequence were mapped on both haplotypes of CL Brener reference genome, Esmeraldo-like and non-Esmeraldo (TriTrypDB v52), using BWA-mem v.0.7.12. The coordinates from which these donor-containing reads mapped were recovered with SAMtools and converted to browser extensible data (BED) format using Awk. Finally, Bedtools intersect (52) with the mapped read coordinates BED file and the gene annotation GFF (general feature format) (TriTrypDB v52) file was used to identify the genes in which the donor-contained reads mapped.

### *In vitro* infections.

Culture-derived trypomastigotes (TCTs) were generated from epimastigotes subjected to nutritional stress, which were added to culture flasks containing LLC-MK2 subconfluent cell monolayers as previously described (20). Parasites were then collected from the supernatants of infected cells from day 5 to 9 and transferred to 50-mL Falcon tubes. The tubes were centrifuged at 1,500 × *g* for 10 min to pellet TCTs and amastigote like forms (as well as cells that might have eventually detached from the flasks and cell debris). Following centrifugation, the tubes were incubated at 34°C for 6 to 12 h, without disturbing the pellets, to allow TCTs swim to the supernatant. Motile TCTs were collected, leaving behind 1 mL of medium to avoid resuspending amastigote-like forms. Purified TCTs were counted and used for protein extraction, mouse infection, or reinfection of subconfluent cell monolayers at a multiplicity of infection (MOI) of 10 trypomastigotes per cell (LLC-MK2), which were adhered to microscope coverslips. The numbers of trypomastigotes released in the supernatant and of intracellular amastigotes were determined as previously described (20). Purified TCTs were also used to evaluate the percentages of infected cells and colocalization of parasites with a lysosomal marker. HeLa cells were infected with parental or TS KO7 cell lines at an MOI of 100. After 1 h of incubation, cells were washed with PBS to remove parasites that failed to enter the cells and fixed with 4% paraformaldehyde 2, 5, and 21 h after infection.

### Mouse infections.

Fifteen female IFN-γ-KO mice were divided into three groups with 5 animals per group and intraperitoneally injected with 10.000 TCTs resuspended in PBS solution. Mouse parasitemia was evaluated on microscopy slides with 5 μL of blood taken from the mouse tail and followed for 40 days, as described before (43). For immunization protocols, eight female BALB/c mice 6 to 8 weeks old were separated into two groups with 4 animals per group and intraperitoneally injected with 5,000 TCTs (TS KO7 parasites). Thirty days after injection, mice were challenged with 10,000 blood trypomastigotes from strain Y, and parasitemia was followed for 30 days. The relative amounts of parasite DNA in various tissues were determined by real-time PCR using TCZ1 and TCZ2 primers as well as primers that amplify the mouse gene for tumor necrosis factor. All animals were kept at the animal facility at the Centro de Pesquisa René Rachou and received food and water *ad libitum*. Experiments involving mice followed protocols approved by the animal care ethics committee of the Federal University of Minas Gerais (CEUA, UFMG) under protocol number 52/2020.

### Antiserum production, Western blotting, and immunofluorescence assays.

For the anti-TS polyclonal antibody (antiserum) production, 6-week-old C57BL/6 female mice were injected three times at 2-week intervals with 10 μg of purified recombinant aTS obtained after cloning of the coding sequence of the gene Tc00.1047053509495.30 into pET21a (Novagen). Expression in E. coli BL21(DE3), after addition of 1 M isopropyl β-d-1-thiogalactopyranoside (IPTG) and purification on a nickel-nitrilotriacetic acid (Ni-NTA) column (GE Healthcare), followed previously described protocols (43). Two weeks after the last injection, the animals were subjected to general anesthesia and whole blood was collected by cardiac puncture with subsequent euthanasia. The sera obtained from collected blood was assessed by enzyme-linked immunosorbent assay (ELISA) with 100 ng of the purified recombinant protein/well, and the serum that produced the highest titer was tested in Western blots with total parasite protein extracts. Protein extracts were prepared from 10^7^ epimastigotes or trypomastigotes and analyzed on Western blots with anti-TS polyclonal antibody diluted 1:1,000 or with antitubulin antibody (1:5,000; Sigma) as previously described (43). Immunofluorescence assays were performed with epimastigotes and trypomastigotes after fixation with 4% paraformaldehyde for 5 min and blocking with 1% bovine serum albumin (BSA), 0.2% Tween 20 for 1 h at room temperature. Parasites were incubated for 1 h with polyclonal anti-TS serum diluted 1:50 or with MAb 3C9 diluted 1:100 followed by incubation with anti-immunoglobulin G (anti-IgG) secondary antibody conjugated to Alexa Fluor 488 (Invitrogen/Life Technologies). After nucleus staining with DAPI (4′,6-diamidino-2-phenylindole; Molecular Probes/Life Technologies), images were acquired with a 63× objective in the Zeiss LSM 880 Airyscan confocal microscope at the Centro de Aquisição e Processamento de Imagens (CAPI-ICB/UFMG). To investigate colocalization of parasites with LAMP-1, cells that had been fixed at different time points after infection (2, 5, and 21 h) were incubated for 20 min at room temperature with blocking buffer (PBS containing 2% bovine serum albumin and 0.5% saponin) followed by incubation with anti-LAMP-1 (MAb H4A4) primary antibody diluted in blocking buffer (1:20) for 45 min at room temperature. After successive washes with blocking buffer and incubation with anti-mouse immunoglobulin conjugated to Alexa Fluor 555 (Life Technologies; A21422; 1:500 dilution) and DAPI (1 μg/mL) to stain nuclei, the images were acquired as described above.

### Flow cytometry assays.

Trypomastigotes were centrifuged at 3,000 × *g* for 10 min, washed once with PBS, and fixed with 4% paraformaldehyde. After being washed once more with PBS, parasites were resuspended in 200 μL of antibody/lectin solution (for MAb 3C9, PBS, 5% BSA; for IB4 isolectin, 1× PBS, 5% BSA, 0.01 M MgCl_2_, 0,01 M CaCl_2_, 0.01 M MnCl_2_) for 1 h. Parasite were then centrifuged at 3,000 × *g* and resuspended in wash solution (for MAb 3C9, 1× PBS, 0.1% BSA, 0.02% sodium azide; for biotinylated IB4, 1× PBS, 0.1% BSA, 0.01 M MgCl_2_, 0.001 M CaCl_2_, 0.01 M MnCl_2_). Parasites were centrifuged at 3,000 × *g* for 5 min and incubated for 30 min in 200 μL incubation solution with Alexa 488 anti-mouse at a 1/200 dilution for MAb 3C9 or streptavidin phycoerythrin at a 1/200 dilution for biotinylated IB4 (Invitrogen). Next, parasites were centrifuged at 3,000 × *g* for 5 min and fixed again with 4% paraformaldehyde. After centrifugation at 3,000 × *g* for 5 min, parasites were resuspended in 500 μL 1× PBS and analyzed on a BD Accuri flow cytometer using the filters FL1 for MAb 3C9 and FL2 for IB4 isolectin.

### Trans-sialidase activity assay.

A total of 2 × 10^7^ TCTs were lysed in an appropriate buffer (50 mM Tris HCl, 0.1 M NaCl, 1% Triton X-100 and Complete C [EDTA-free protease inhibitor; Roche Diagnostics]), and different amounts of sample (1 μL, 2.5 μL, 5.0 μL, and 10.0 μL) were incubated with fetal bovine serum, 1% BSA, HEPES, and d-glucose–[^14^C]lactose (New England Nuclear) for 30 min at room temperature. Then, the reaction products were purified using a QAE-Sephadex column, and the activities of purified products were measured in counts per minute in a scintillation counter (Tri-Carb 2810 TR; Perkin Elmer).

### Antibody and cytokine measurements.

To evaluate the antibody response, ELISA was performed in 96-well microtiter plates (Nunc Immunoplates) coated with T. cruzi protein extracts (diluted at 20 μg/mL in 100 μL of carbonate buffer per well; pH 9.6). Mouse sera diluted 1:100 in blocking buffer (PBS, 0.05% Tween 20, and 3% low-fat milk) were incubated for 1 h at 37°C. Peroxidase-conjugated goat anti-mouse IgG, IgG1, and IgG2a (SouthernBiotech) diluted 1:1,000 were incubated for 1 h at 37°C, and peroxidase reactions were detected with 1 mg/mL 3,3′,5,5′-tetramethylbenzidine (TMB; Sigma-Aldrich). Optical density was measured spectrophotometrically at 450 nm. To evaluate the cellular immune response, animals were euthanized 20 days after infection, and splenocyte cultures were performed as described previously (43). IFN-γ was measured in supernatants by ELISAs using R&D Systems kits, following the manufacturer’s instructions.
